# Regional disparity of HIV incidence and prevalence among men who have sex with men

**DOI:** 10.1186/s12879-021-06582-x

**Published:** 2021-09-06

**Authors:** D. N. Vergara-Ortega, H. López-Gatell, S. Bautista-Arredondo, A. Colchero, S. G. Sosa-Rubí, M. Morales-Vazquez, A. Herrera-Ortiz, M. Olamendi-Portugal, S. García-Cisneros, E. E. Sevilla-Reyes, M. Hernández-Avila, M. A. Sánchez-Alemán

**Affiliations:** 1 Centro de Investigación Sobre Enfermedades Infecciosas, Instituto Nacional de Salud Pública, Av. Universidad No.655, Col. Santa María Ahuacatitlán, 62100 Cuernavaca, Morelos Mexico; 2 Centro de Investigación en Sistemas de Salud, Instituto Nacional de Salud Pública, Cuernavaca, Morelos Mexico; 3 Instituto Nacional de Enfermedades Respiratorias, Mexico City, Mexico; 4 Centro Universitario de Ciencias de la Salud, Universidad de Guadalajara, Guadalajara, Mexico

**Keywords:** Incidence, Prevalence, HIV, HIV transmission, Risk factors, HIV MSM

## Abstract

**Background:**

HIV incidence can be estimated with cross-sectional studies using clinical, serological, and molecular data. Worldwide, HIV incidence data in only men who have sex with men (MSM) are scarce and principally focus on those with healthcare or under treatment. However, better estimates can be obtained through studies with national representativeness. The objective was to estimate the prevalence, incidence, and factors associated with acquiring HIV in a national sample of MSM who attend meeting places, considering geographical regions.

**Methods:**

A nationally representative survey of MSM attending meeting places was performed in Mexico. Participants answered a questionnaire, and a dried blood spot (DBS) was collected. Samples were classified as recent infections using an algorithm with HIV status, antiretroviral therapy, and the result of BED-EIA assay. Parameters were analysed considering regions and demographic and sexual behaviour characteristics.

**Results:**

The national HIV prevalence was 17.4% with regional differences; the highest prevalence (20.7%) was found in Mexico City, and the lowest prevalence was found in the West region (11.5%). The incidence was 9.4 per 100 p/y, with regional values from 6.2 to 13.2 for the Northeast and the Centre regions, respectively. Age, age at sexual debut, low wealth index, and rewarded sex were associated with HIV prevalence. Centre region, use of private clinics as health services, and having sex exclusively with men were associated with recent HIV infections.

**Conclusions:**

The incidence and prevalence showed regional differences, suggesting a difference in the dynamics of HIV transmission; some regions have a greater case accumulation, and others have a greater rate of new infections. Understanding this dynamic will allow developing health programs focused on HIV prevention or treating people already living with HIV.

## Background

In 2019, approximately 38 million people were living with HIV (PLWH) worldwide, with 1.7 million new HIV infections representing a 40% reduction relative to 1998 (2.8 million) [1]. However, the incidence in vulnerable populations, such as men who have sex with men (MSM), has increased. MSM have up to 13 times higher risk of acquiring HIV than other population groups [1, 2]. Mexico, a country with 127 million inhabitants distributed in 32 states, has a concentrated HIV epidemic. According to the National Health and Nutrition Survey 2012 (ENSANut 2012), the HIV seroprevalence in the general population was 0.15%, while in key population groups, it was higher ranging from 12 to 15.1% among male sex workers, 7% among intravenous drug users [3], and 16.9% among MSM [4].

Prevalence and incidence estimation reflect the magnitude of the epidemic. To calculate HIV incidence through transversal studies, the World Health Organization (WHO) has recommended the design of the recent infection test algorithm (RITA) using clinical information, serological tests, and molecular assays. In addition to the algorithm, the use of a correction factor is recommended. Regardless of the type of study, a better estimation would be obtained with a nationally representative sample [5].

There is little information on HIV incidence in population-based and nationally representative studies, and it is even more scarce in vulnerable populations such as MSM with information based only on populations in health care and/or monitoring, specifically in people who had contact with health services [6–8].

In 2011, a nationally representative survey focused on HIV seroprevalence was conducted in Mexico among MSM at meeting places. The HIV prevalence among 7,823 participants with a point-of-care test was 16.9% [4, 9]. The results showed that the risk of HIV infection increased with age, the number of sexual partners, a receptive sexual role, and low educational level [9]. In 2013, the second version of this survey was carried out as an instrument to evaluate the prevention programs in relation to the first survey from 2011. This survey included similar meeting place and a similar questionnaire (about demographics, sexual behaviours, lifestyle, and health care), and a DBS was collected for each participant [10]. The objectives of the current study were (1) to estimate HIV prevalence and incidence among participants in the 2013 survey and (2) to estimate sociodemographic and behavioural factors associated with HIV prevalence and recent HIV infections, considering the differences between geographical regions.

## Methods

### Survey design and fieldwork

The national survey of MSM attending meeting places was performed between September and November 2013 in 24 states distributed in 6 geographic regions: Mexico City, South, Centre, Northeast, Northwest, and West. These cities were selected based on their population size and HIV prevalence from an initial group of 44 cities with the greatest prevalence reported between 2003 and 2008 [4, 10]. The sample size of each region was based on the census that was carried out prior to this work to determine an approximate size of the total population of MSM who attend the meeting places and to determine the access to health services of MSM related to a previous intervention [4, 10].

An adaptation of the PLACE method was used to identify the MSM meeting places, and these were randomized to recruit the participants at convenience. The selected meeting places were visited by the interviewers who asked for other places, and they followed the same procedure until a saturation point was reached [10]. After signing the informed consent form, each participant answered an anonymous self-reported electronic questionnaire (using the ACASI system). The questionnaire took between 30 and 40 min to complete. DBS samples were collected by trained nurses. The inclusion criteria were being MSM, being over 18 years of age, providing written informed consent, and answering the questionnaire. The exclusion criteria were incomplete information and insufficient biological samples. All the men in the study voluntarily agreed to participate without any kind of compensation. The study population for our study was 6840 MSM surveyed in 2013 [10].

### Laboratory tests

Detection of HIV cases was performed with a chemiluminescent microparticle immunoassay by simultaneous detection of antigens and antibodies with DBS samples (HIV Ag/Ab Combo, Architect, Abbott Diagnostics Division™). To identify possible recent HIV infections, a serological test of differences in proportions was performed following the manufacturer’s instructions (BED EIA HIV-1, Sedia™). To classify recent infections, we first generated an algorithm that included the HIV Ag/Ab Combo assay, information about antiretroviral therapy (ART), and BED EIA HIV-1 test results. To estimate the incidence, we used recent HIV infections and the formula proposed by Hargrove et al. with a 1.3% correction factor (FRR) previously obtained for MSM from Mexico City for DBS samples and the BED EIA HIV-1 test [11, 12].

### Statistical analyses

The cities were grouped into six regions: (1) Mexico City, (2) South (Merida, Cancun, and Campeche), (3) Centre (Puebla, Tlaxcala, Cuernavaca, Tehuantepec-Juchitan, Veracruz, Acapulco, Xalapa, and Pachuca), (4) Northeast (Monterrey, San Luis Potosi, Reynosa-Rio Bravo, and Matamoros), (5) Northwest (Tijuana, Juarez, Mexicali, and Hermosillo), and (6) West (Guadalajara, Leon-Silao, Aguascalientes, and Puerto Vallarta). The “wealth index” was classified as low (0–2 goods), medium (3–4 goods), and high (5–7 goods) based on 7 goods: refrigerator, washing machine, water heater, computer, internet, domestic employees, and automobiles. “Health services” refers to the site at which the participant looks after his medical events in the last year. “HIV test offered” refers to a number of tests offered to the participant in the last year, and “HIV test” refers to the site where the participant performed the HIV test in the last year. “Age of sexual debut” classifies the age of the participant at the time of their first sexual intercourse. “Sexual partners’ gender” considers sexual intercourse with men only or with men and women. “Rewarded sex” refers to receiving money or gifts for sex. The “last sexual partner’s HIV status” describes the knowledge of the HIV status of the partner. “Known people with HIV/AIDS” refers to acquaintances infected with HIV or suffering from AIDS. “Number of sexual partners” classifies the number of all sexual partners in the last month. “Condom use” was defined as the consistency of condom use in the last three sexual intercourses. “Sexual role” refers to the insertive or receptive role in the last three sexual intercourses. A descriptive analysis was developed by geographic region with 95% confidence intervals (95% CI). The results of HIV status (negative, prevalent, or recent infections) gave rise to the dependent variables used in the analysis. A bivariate multinomial logistic regression of prevalence and recent HIV infections was carried out. The logistic regression began with a bivariate analysis, and the variables with a p < 0.05 and the sexual partners variables were analysed in a saturated model. The variables that were not statistically significant were eliminated, except for the sexual partners variable because of its relevance in HIV transmission. The backward step-by-step method was used to perform a multivariate logistic regression. To analyse the dynamics of HIV for each geographic region, we created the following regional variables: “HIV diagnosis percentage” with the total number of participants who knew their HIV status and the total laboratory-confirmed HIV positives, “antiretroviral treatment (ART) coverage” considering the number of MSM with ART and the total number of HIV-positive individuals who knew their status, and “mortality” (weighted percentage of HIV mortality) with information from an external study published by Bravo-García and Ortíz-Pérez [13]. The statistical analyses were performed using SPSS Statistics version 25.0 (IBM).

### Ethics statement

All the methods in the study were performed under the guidelines, regulations, and instructions of ethics and biosafety *Instituto Nacional de Salud Pública’s* committees. After the complete observation and evaluation of the study by the committees, we obtained approval from both ethics 1393 and biosafety 1002. Each participant read and signed the informed consent form.

## Results

Most of the participants were between 21 and 30 years old, with the highest percentage living in the South (60.7%) and the lowest in the Northwest (46.8%). Regarding the wealth index, the percentage of persons in the highest stratum was greatest in Mexico City (70.6%) and the lowest in the South (58.7%). Regarding the sexual behaviour variables, 37.6% of the MSM reported that their last sexual partner did not have HIV, with regional differences ranging from 34.1% in Mexico City to 48.3% in the South. For the sexual role, 25.8% reported being sexually insertive in the last three sexual relationships, and the greatest differences were found between the Northwest (29.1%) and Centre (22.9%). The population presented differences by geographic region in both sociodemographic and sexual behavioural characteristics, as shown in Table 1.

**Table 1:** Demographic and sexual behavior characteristics among men who have sex with men, stratified by region

Variable	Categories	Total %	Region (%)						p value
			Mexico city	South	Centre	Northeast	Northwest	West	
Age (years)	≥ 41	9.2	9.2	6.0	5.2	14.6	15.2	5.0	< 0.001^&^
	31–40	18.8	19.8	10.7	14.6	22.8	23.4	15.7	
	21–30	54.0	54.6	60.7	55.5	48.9	46.8	58.1	
	18–20	18.0	16.4	22.7	24.7	13.6	14.5	21.2	
Wealth index	Low	10.5	9.0	19.3	11.4	11.6	13.8	8.8	< 0.001^&^
	Medium	22.0	20.4	22.0	24.7	25.3	23.5	21.0	
	High	67.4	70.6	58.7	63.9	63.2	62.7	70.2	
Health service	HIV Specialized Clinic	1.0	1.0	1.0	1.1	1.2	1.1	0.4	< 0.001^&^
	Government	16.1	17.2	19.3	13.1	14.0	11.9	19.2	
	Private	12.4	13.4	11.7	14.7	9.5	8.8	11.6	
	No service used	70.6	68.3	68.0	71.0	75.4	78.2	68.8	
HIV test offered	≥ 6	3.2	3.2	2.7	1.3	2.4	4.9	5.1	< 0.001^&^
	1–5	34.9	37.5	33.0	26.8	27.5	40.0	37.5	
	None	61.9	59.4	64.3	71.9	70.1	55.1	57.4	
HIV test	Government	29.3	31.4	30.3	25.6	24.6	33.1	26.8	< 0.001^&^
	Private	12.5	12.0	15.3	12.0	13.0	13.1	12.8	
	No test	51.9	50.9	47.7	55.9	56.4	48.1	51.8	
	Meeting points	6.3	5.7	6.7	6.4	6.0	5.7	8.6	
Age of sexual debut with men	8–14	21.7	22.9	23.0	19.4	20.7	22.4	20.2	< 0.001^&^
	15–19	55.5	54.9	59.0	58.9	52.0	51.6	59.1	
	≥ 20	22.7	22.2	18.0	21.7	27.3	26.0	20.7	
Sexual partners gender	Men only	87.4	89.0	85.0	86.4	83.1	82.6	91.3	< 0.001^&^
	Men and women	12.6	11.0	15.0	13.6	16.9	17.4	8.7	
Rewarded sex	Yes	26.7	26.7	35.3	24.0	26.6	28.0	25.9	0.007^&^
	No	73.3	73.3	64.7	76.0	73.4	72.0	74.1	
Last sexual partner HIV status	I know he has HIV	4.0	5.0	3.0	2.8	3.0	3.9	3.3	< 0.001^&^
	I don’t know if he has HIV	38.4	40.4	33.7	35.8	37.6	37.4	37.4	
	I think he does not have HIV	20.0	20.5	15.0	19.5	20.5	19.4	20.4	
	I know he does not have HIV	37.6	34.1	48.3	41.8	38.9	39.3	38.9	
Known people with HIV/AIDS	Yes	55.2	61.6	56.3	46.9	46.2	55.1	49.6	< 0.001^&^
	No	44.8	38.4	43.7	53.1	53.8	44.9	50.4	
Number of sexual partners	No answer	7.0	7.1	9.7	9.4	3.7	6.7	6.1	< 0.001^&^
	≥ 11	1.7	2.3	1.0	1.3	0.9	1.3	1.3	
	2–10	27.2	29.3	28.7	25.6	23.4	26.3	24.9	
	0–1	64.1	61.3	60.7	63.6	72.0	65.7	67.7	
Condom use	No answer	5.9	6.0	8.3	6.9	2.8	6.3	6.0	< 0.001^&^
	None	16.1	14.4	22.0	18.8	15.9	18.9	15.5	
	1–2 times	16.5	16.1	16.7	15.5	15.0	17.8	19.2	
	3 times	61.5	63.6	53.0	58.9	66.2	57.0	59.3	
Sexual role	No answer	6.3	6.4	8.7	7.3	3.2	7.2	6.4	< 0.001^&^
	Receptive	21.3	20.8	23.7	22.2	23.4	22.1	18.8	
	Both	46.6	47.5	40.0	47.6	44.5	41.6	50.3	
	Insertive	25.8	25.3	27.7	22.9	28.9	29.1	24.5	

The table shows the population description of MSM who gather at meeting points (n = 6840) by region. ^&^Statistically significant (p-value < 0.001, obtained with Chi-squared tests)

**Fig. 1:**
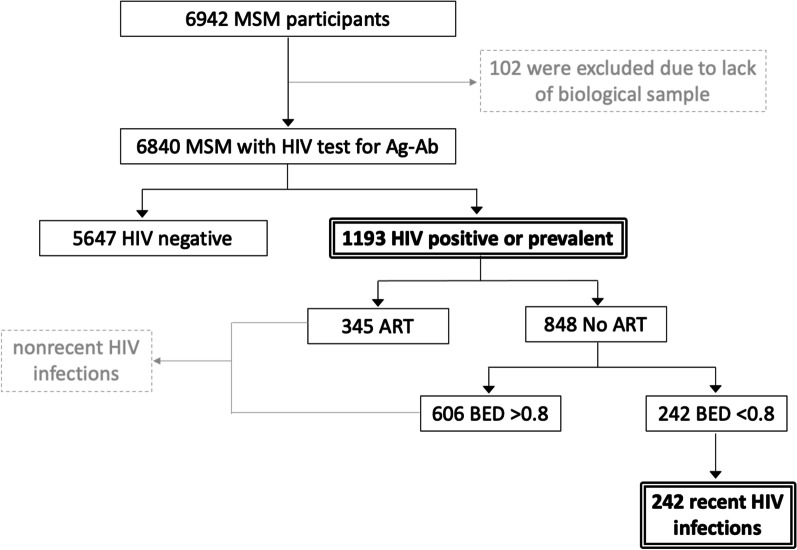
Recent infection test algorithm (RITA). It shows the designed algorithm that combines the results of serological tests and information on the initiation of ART (antiretroviral therapy), each step with its corresponding number of samples. The 242 recent HIV infections were used to calculate the incidence

**Fig. 2:**
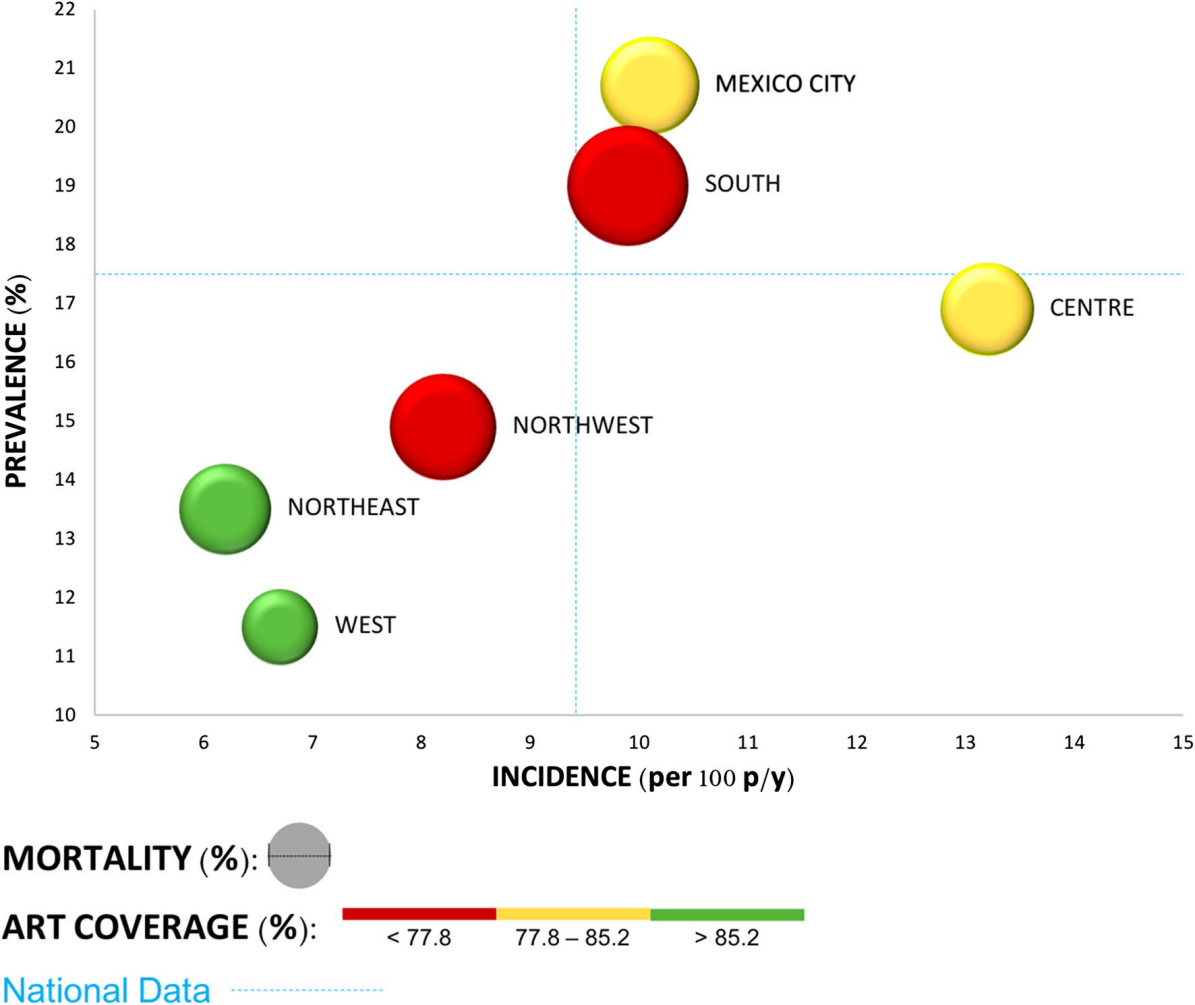
Scenarios of HIV transmission by geographic region. Based on the regional variables, we constructed the graph concerning national data (blue lines); located each geographical region in terms of its prevalence and incidence. With this organization, we observed three regions (West, Northeast and Northwest), lean towards the lower left quadrant, which can be considered the one with the best situation because of its lowest prevalence and incidence. On the contrary, South, Mexico City and Centre, are located towards the part of the graph where the highest prevalence and incidence data are displayed. Likewise, mortality corresponds with the diameter of the bubbles and the coverage of antiretroviral treatment with the color

The study began with 6942 MSM; 102 were excluded due to lack of DBS samples, 1193 were positive for HIV testing (HIV Ag/Ab Combo), 848 reported no ART among which 242 were classified as recent HIV infections by the BED EIA HIV-1 assay (Fig. 1). The national HIV prevalence was 17.4% (95% CI 16.6–18.4), with differences between regions; the highest prevalence was in Mexico City, at 20.7%, and the lowest was in the West, at 11.5%. The national incidence was calculated at 9.4 per 100 person/years (p/y) (95% CI 4.4–7.9), with the Centre having the highest incidence at 13.2 per 100 p/y and the Northeast having the lowest incidence at 6.2 per 100 p/y. The geographic regions showed disparities; for example, the highest percentage of diagnosis was found in Mexico City (41.2%), and the lowest percentage was found in the Centre (22.1%). The ART coverage was 81.8% at the national level, with differences from 77.3% in the South to 90.9% in the Western, and the mortality ranged from 7.0 in the South and 2.8 in the West (Table 2 and Fig. 2).

**Table 2:** Regional HIV prevalence and incidence among men who have sex with men

Region	Cities	n	Prevalence (%)	Incidence (per 100 p/y)	Diagnosis (%)	Art coverage (%)	*Mortality (%)
Mexico city	Mexico City	3162	20.7	10.1	41.2	80.7	4.6
South	Merida, Cancun, and Campeche	300	19.0	9.9	38.6	77.3	7.0
Centre	Puebla-Tlaxcala, Cuernavaca, Tehuantepec-Juchitan, Veracruz, Acapulco, Xalapa, and Pachuca	963	16.9	13.2	22.1	80.6	4.1
Northeast	Monterrey, San Luis Potosi, Reynosa-Rio Bravo, and Matamoros	779	13.5	6.2	28.6	90.0	4.0
Northwest	Tijuana, Juarez, Mexicali, and Hermosillo	697	14.9	8.2	29.8	77.4	5.4
West	Guadalajara, Leon-Silao, Aguascalientes, and Puerto Vallarta	939	11.5	6.7	30.6	90.9	2.8
National		6840	17.4	9.4	35.4	81.8	4.2

Prevalence, diagnosis, antiretroviral therapy (ART) coverage, and mortality columns show the data calculated for each region in percentage (%); incidence represents the regional data per 100 persons/years (p/y). *Mortality was calculated with data obtained from external information [13]

Regarding the factors associated with HIV prevalence, from adjusted analyses, we found that MSM in the South were two times more likely to be infected than people in the West. The HIV prevalence increased with age and among people with a low wealth index. Being treated in an HIV specialized clinic was associated with the highest HIV prevalence, and these individuals were 12.4 times more likely to live with HIV than people without this service. Regarding sexual behaviour, individuals with early sexual debut with men (8–14 years) had a 1.6 times higher likelihood of HIV infection than individuals with sexual debut at or after age 20; men who had sex only with men had a 1.8 times higher probability of living with HIV than men who had sex with men and women. Those who knew that their last sexual partner had HIV were almost 5 times more likely to have HIV infection. Other variables, such as HIV test offered, HIV test, rewarded sex, knowing individuals with HIV/AIDS, condom use (none and 1–2 times), and sexual role, were associated with HIV prevalence, as shown in Table 3.

**Table 3:** Sociodemographic and sexual behavior variables and their association with HIV prevalence and recent HIV infections

Variable	Categories	Prevalence (%)	Prevalence OR_A_ (CI _95%_)	Recent infections (%)	Recent infections OR_A_ (CI _95%_)
Region	Mexico City	20.7	1.6 (1.2–2.0)^&^	3.7	1.5 (0.9–2.3)
	South	19.0	1.9 (1.2–2.8)^&^	3.7	1.6 (0.8–3.3)
	Centre	16.9	1.9 (1.4–2.6)^&^	5.0	2.2 (1.3–3.6)^&^
	Northeast	13.5	1.3 (0.9–1.8)	2.4	1.0 (0.6–1.9)
	Northwest	14.9	1.2 (0.9–1.7)	3.2	1.3 (0.7–2.3)
	West	11.5	1	2.7	1
Age (years)	≥ 41	22.2	2.2 (1.6–3.1)^&^	4.1	1.2 (0.8–1.9)
	31–40	23.0	2.2 (1.7–3.0)^&^	3.4	1.5 (0.9–2.5)
	21–30	17.4	1.7 (1.3–2.1)^&^	3.5	1.1 (0.8–1.6)
	18–20	9.4	1	3.4	1
Wealth index	Low	21.8	1.7 (1.3–2.1)^&^	3.8	1.2 (0.8–1.8)
	Medium	17.5	1.1 (0.9–1.3)	3.1	0.9 (0.6–1.2)
	High	16.7	1	3.6	1
Health service	HIV Specialized Clinic	75.0	12.4 (6.2–24.9)^&^	5.9	4.9 (1.6–15.2)^&^
	Government	28.2	2.1 (1.7–2.5)^&^	3.5	1.2 (0.8–1.8)
	Private	20.6	1.6 (1.3–2.0)^&^	5.0	1.6 (1.1–2.2)^&^
	No service used	13.6	1	3.3	1
HIV test offered	≥ 6	25.7	1.7 (1.1–2.5)^&^	4.5	1.5 (0.7–2.9)
	1–5	20.5	1.4 (1.2–1.7)^&^	3.9	1.2 (0.9–1.6)
	None	15.3	1	3.3	1
HIV test	Government	23.1	2.4 (1.7–3.5)^&^	3.7	1.2 (0.7–2.0)
	Private	16.6	2.0 (1.3–2.9)^&^	4.2	1.3 (0.7–2.4)
	No test	15.1	2.3 (1.6–3.4)^&^	3.3	1.2 (0.7–2.1)
	Meeting points	11.9	1	3.7	1
Age of sexual debut with men	8–14	23.1	1.6 (1.3–2.1)^&^	3.7	1.2 (0.8–1.9)
	15–19	16.5	1.4 (1.1–1.7)^&^	3.8	1.3 (0.9–1.9)
	≥ 20	14.4	1	2.8	1
Sexual partners gender	Men only	18.6	1.8 (1.3–2.4)^&^	3.8	2.1 (1.2–3.6)^&^
	Men and women	9.3	1	1.7	1
Rewarded sex	Yes	22.1	1.3 (1.1–1.5)^&^	4.0	1.1 (0.8–1.5)
	No	15.7	1	3.4	1
Last sexual partner HIV status	I know he has HIV	46.9	4.4 (3.2–6.0)^&^	1.8	0.7 (0.3–1.8)
	I do not know if he has HIV	21.0	1.6 (1.4–2.0)^&^	4.1	1.2 (0.9–1.6)
	I think he does not have HIV	14.0	1.1 (0.8–1.3)	2.9	0.8 (0.5–1.2)
	I know he does not have HIV	12.5	1	3.5	1
Known people with HIV/AIDS	Yes	23.9	2.2 (1.8–2.6)^&^	4.1	1.5 (1.2–2.1)^&^
	No	9.5	1	2.8	1
Number of sexual partners	No answer	20.3	1.1 (0.7–1.5)	4.0	0.9 (0.5–1.7)
	≥ 11	23.9	0.9 (0.5–1.4)	1.7	0.4 (0.1–1.8)
	2–10	19.9	1.1 (0.9–1.2)	4.4	1.3 (1.0–1.7)
	0–1	15.9	1	3.2	1
Condom use	No answer	18.7	0.2 (0.1–0.5)	5.0	1.2 (0.4–3.3)
	None	10.3	0.5 (0.4–0.7)^&^	1.9	0.5 (0.3–0.7)^&^
	1–2 times	15.8	0.7 (0.6–0.9)^&^	3.6	0.9 (0.6–1.2)
	3 times	19.6	1	3.8	1
Sexual role	No answer	20.6	3.3 (1.8–6.2)^&^	4.6	2.0 (0.7–5.9)
	Receptive	19.8	1.6 (1.3–2.0)^&^	4.6	2.2 (1.4–3.3)^&^
	Both	18.3	1.6 (1.3–1.9)^&^	3.7	1.8 (1.3–2.7)^&^
	Insertive	13.1	1	2.1	1

The table shows the sociodemographic and sexual behaviour variables and their association with HIV prevalence or recent HIV infections. The odds ratio (OR_A_) was adjusted for all the following variables: region, age, wealth index, health service, HIV test offered, HIV test, age of sexual debut, sexual partners gender, rewarded sex, last sexual partner HIV status, known people with HIV/AIDS, condom use, and sexual role. ^&^Statistically significant (p-value < 0.05, obtained with Chi-squared tests)

In relation to the factors associated with recent HIV infection with the adjusted model, individuals in the Centre were 2.2 times more likely to have HIV in comparison with those in the West. Older age, early age at sexual debut, and low wealth index were not associated with recent infection. Men who had sex only with men were twice as likely to acquire HIV than men who had sex with men and women. MSM only who reported a receptive role were 2.2 times more likely to have a recent infection than MSM only who reported an insertive role. MSM who attended HIV specialized clinics were approximately five times more likely to have acquired HIV infection than MSM without these services. Individuals who knew a person with HIV had a 1.5 times higher probability of having a recent infection. The consistent use of condoms was not found to be a protective factor for HIV, and the number of sexual partners in the last month did not show differences in likelihood of recent HIV infection, as shown in Table 3.

## Discussion

The national HIV prevalence found among MSM in Mexico (17.4%) was higher than the prevalence in the general male population (0.24%) [3] but similar to that in the MSM population from Sub Saharan African (17.9%), less than that in Caribbean countries (25.4%), and higher than that in others global regions (Central and South America 14.9% and North America 15.4%) [14]. However, across the country, the HIV prevalence in MSM is heterogeneous, ranging from 11.5% in the West region to 20.7% in Mexico City. Regarding HIV incidence, there is limited information about vulnerable groups (such as MSM) in national surveys. The incidence reported in our study in the MSM population in Mexico (9.4 cases per 100 p/y) was higher than that obtained for the general population (0.100–0.250 per 1000 p/y) [15] and the Central Latin American region (0.0943 per 1000 p/y) [14]. Compared to the incidence calculated in small MSM groups in the Latin American countries of Ecuador (6.5 cases per 100 p/y), Peru (3.5 cases per 100 p/y), and Brazil (5.0 cases per 100 p/y) [16], our results showed the highest incidence. Nevertheless, our data are similar to areas with a concentrated epidemic, showing 7.4 cases per 100 p/y among young MSM from Bangkok [17], and countries with a generalized epidemic, such as 12.5 cases per 100 among MSM in South Africa [18].

The current study shows the HIV incidence from MSM at the national and regional levels, and this data was not collected only from local surveys or by only including the general population. For this work, we considered the use of the RITA algorithm and a specific FRR for correction, as recommended by the World Health Organization [5, 12]. A publication by Hallett T. in 2011 showed that none of 39 studies analysed corrected the results or data obtained from other populations or regions by a specific FRR; consequently, the incidence estimations may contain errors [19].

Our results show the contrasts between the prevalence and the estimated incidence at a regional level, indicating that the population dynamics of the HIV epidemic among MSM differ within the same country. The transmission dynamics could be influenced by multiple variables at different levels with different impacts [20]. The West, Northeast, and Northwest regions stand out with the lowest prevalence. The first two regions showed high ART coverage and low mortality, which are regional variables that could explain their low prevalence and incidence. In summary, the better the treatment is, the lower the transmission (low population viral load) and mortality [21]. On the other hand, in the Northwest, the low estimates may reflect low ART coverage and high mortality [13, 21]. Therefore, the health status of the newly infected population must be affected by poor treatment, low survival, and therefore low case accumulation. The low prevalence of HIV in the West region is associated with a high socioeconomic level, late initiation of sexual activity, and having an insertive sexual role, individual characteristics that are found in a high percentage in the West region and that are associated with decreased HIV prevalence. The Northeast region had the lowest incidence of HIV, which can be explained in part because it has the highest percentage of people with a late sexual debut, a lower percentage of people who have relationships only with men, the lowest percentage of people who know someone with HIV, and the lower percentage of receptive MSM, which are protective factors against HIV incidence.

In contrast, the South, Mexico City, and Centre regions presented the highest prevalence and incidence. The estimates for the South are explained by its high mortality (the highest of all regions) [13] and its very low ART coverage. Poor treatment results in greater transmission, more cases, and higher mortality [8, 21]. The high prevalence and incidence found in the Mexico City and Centre regions can be explained by the relatively low ART coverage; it causes an accumulation of cases (high prevalence) and does not reduce the viral load, resulting in conditions of sustained transmission of the virus (high incidence) [8, 21]. Finally, comparing the Mexico City and Centre regions, the difference in prevalence could be explained by the highest percentage of diagnosis in the Mexico City (41.2%) and the lowest in the Centre region (22.1%). The heterogeneity between regions shows that the prevalence and incidence are insufficient to explain the dynamics of HIV transmission, which is why there are different epidemics in the same country. The high prevalence of HIV in Mexico City is related to individual characteristics such as early sexual debut, knowing that the last sexual partner had HIV, meeting people with HIV, and having only male sexual partners. These variables were associated with HIV prevalence and were found to be more frequent in Mexico City. In the Centre region, there is a high percentage of MSM with receptive sexual roles, which is a risk factor at the individual level and contributes to the incidence of HIV in this region.

At the individual level, time-dependent variables such as age or age at sexual debut were associated with HIV prevalence because these variables indicate a longer period of exposure [22–24]. The HIV status of the last sexual partner was also associated with HIV prevalence, possibly by serosorting, or when people seek sex with those of the same HIV status. In agreement with other publications, MSM who knew that their last sexual partner was living with HIV were approximately 5 times more likely to be infected [25]. For recent HIV infections, we found an association with knowing people living with HIV, and this variable could indicate greater awareness of taking the HIV diagnostic test or the perception of having risky sexual behaviours [26]. The use of HIV specialized clinics was associated with recent HIV infections; in this sense, the Centers for Disease Control and Prevention (CDC) recommend that MSM should be tested for HIV at least once a year [26], so the likelihood of receiving HIV diagnosis increases. The inconsistent use of a condom was apparently a protective factor against HIV acquisition; although this is counterintuitive, it is explained by the reverse causality of condoms being used after HIV is acquired. MSM living with HIV used more condoms once they knew their status [22, 27, 28]. A study in India showed that women who have used condoms had a 3.3 times higher risk of having HIV than women who have never used condoms [29]. Another point to consider is risk compensation: MSM with a low number of sexual partners perceive a low risk of acquiring HIV, and they reduce their condom use. In this study, 81.6% of MSM with fewer sexual partners (0–1) reported never wearing condoms, in contrast to 17.1% and 1.3% of MSM with 2–10 and 11 or more sexual partners, respectively.

The results of this paper should be considered in light of the following limitations. The sexual risk behaviour data were self-reported, and these sensitive behaviours may have been underreported. Additionally, the recruitment strategy of our population (we recruited MSM gathering at meeting places such as discos, bars, cinemas, and public squares) may result in a selection bias leading to high-risk subpopulation groups and may not represent the entire MSM population. Finally, the mortality data used in the analyses come from another study carried out by Bravo-García in the general population [13]. This is because it was impossible to assess mortality (cross-sectional design, self-reported data) in our study population.

## Conclusion

According to the findings of this work, HIV transmission can be addressed at different levels. The effect of regional variables can explain the regional disparity in HIV prevalence and incidence, determine different scenarios of HIV transmission, and hence highlight the different dynamics of the HIV epidemic within the same country. Although Mexico, in general, is classified as a country with an HIV epidemic concentrated in groups with risk behaviours, the variables analysed at both the individual and population levels contribute to this dynamic with different impacts in each region, which leads to an epidemic with specific dynamics in each region analysed.

Therefore, clarifying and understanding these dynamics would allow health programs to focus on the prevention of HIV or on the treatment of infected people, per its transmission dynamics and its specific geographic region. Future research is required with different approaches to understand and clarify factors that could explain the regional disparity in the prevalence and incidence of HIV/AIDS among MSM.

## Data Availability

The datasets used and/or analysed during the current study are available from the corresponding author upon reasonable request.

## Abbreviations

HIV: Human Immunodeficiency Virus
MSM: Men who have sex with men
DBS: Dried blood spot
p/y: Person/years, units of incidence
PLWH: People were living with HIV
ENSANut: From the Spanish of National Health and Nutrition Survey
WHO: World Health Organization
RITA: Recent infection test algorithm
ACASI: Audio computer-assisted self-interview
Ag: Antigen
Ab: Antibody
ART: Antiretroviral therapy
FRR: Correction factor
CDC: Centers for Disease Control and Prevention
AIDS: Acquired Immunodeficiency Syndrome
OR: Odds ratio

## References

[1] Global HIV & AIDS statistics—Fact sheet [01/15/2021]. https://www.unaids.org/es/resources/fact-sheet.

[2] AIDSinfo I UNAIDS [Internet]. [citado 25 de julio de 2021]. Disponible en: https://aidsinfo.unaids.org/.

[3] Gutiérrez JP, Sucilla-Pérez H, Conde-González CJ, Izazola JA, Romero-Martínez M, Hernández-Ávila M (2014). Seroprevalencia de VIH en población mexicana de entre 15 y 49 años: resultados de la Ensanut 2012. Salud Publica Mex.

[4] Bautista-Arredondo S, Colchero MA, Romero M, Conde-Glez CJ, Sosa-Rubí SG (2013). Is the HIV epidemic stable among MSM in Mexico? HIV prevalence and risk behavior results from a nationally representative survey among men who have sex with men. PLoS ONE.

[5] World Health Organization. When and how to use assays for recent infection to estimate HIV incidence at a population level. World Health Organization. 2011. https://apps.who.int/iris/handle/10665/44612.

[6] Kay ES, Batey DS, Mugavero MJ (2016). The HIV treatment cascade and care continuum: updates, goals, and recommendations for the future. AIDS Res Ther.

[7] National Center for HIV/AIDS, Viral Hepatitis, STD, and TB Prevention. Division of HIV/AIDS Prevention. Guidance on community viral load: a family of measures, definitions, and method for calculation. Division of HIV/AIDS Prevention. National Center for HIV/AIDS. 2011. https://stacks.cdc.gov/view/cdc/28147ER.

[8] Miller WC, Powers KA, Smith MK, Cohen MS (2013). Community viral load as a measure for assessment of HIV treatment as prevention. Lancet Infect Dis.

[9] Bautista-Arredondo S, Colchero A, Sosa-Rubí SG, Romero-Martínez M, Conde-González C. Resultados principales de la encuesta de sero-prevalencia en sitios de encuentro de hombres que tienen sexo con hombres. https://www.insp.mx/images/stories/Centros/CIEE/121115_resultadosEncuestaHSH.pdf.

[10] Colchero MA, Bautista-Arredondo S, Cortés-Ortiz MA, Romero-Martinez M, Salas J, Sosa-Rubí SG (2016). Impact and economic evaluations of a combination prevention programme for men who have sex with men in Mexico. AIDS Lond Engl.

[11] Hargrove JW, Humphrey JH, Mutasa K, Parekh BS, McDougal JS, Ntozini R (2008). Improved HIV-1 incidence estimates using the BED capture enzyme immunoassay. AIDS.

[12] Karatzas-Delgado EF, Ruiz-González V, García-Cisneros S, Olamendi-Portugal ML, Herrera-Ortiz A, López-Gatell H (2020). Evaluation of an HIV recent infection testing algorithm with serological assays among men who have sex with men in Mexico. J Infect Public Health.

[13] Bravo-García E, Ortiz-Pérez H (2016). Analysis of HIV/AIDS mortality in Mexico from 1990 to 2013: an assessment of the feasibility of millennium development goals by 2015. Gac Med Mex.

[14] Murray CJL, Ortblad KF, Guinovart C, Lim SS, Wolock TM, Roberts DA (2014). Global, regional, and national incidence and mortality for HIV, tuberculosis, and malaria during 1990–2013: a systematic analysis for the Global Burden of Disease Study 2013. The Lancet.

[15] Frank TD, Carter A, Jahagirdar D, Biehl MH, Douwes-Schultz D, Larson SL (2019). Global, regional, and national incidence, prevalence, and mortality of HIV, 1980–2017, and forecasts to 2030, for 195 countries and territories: a systematic analysis for the Global Burden of Diseases, Injuries, and Risk Factors Study 2017. Lancet HIV.

[16] Luz PM, Veloso VG, Grinsztejn B (2019). The HIV epidemic in Latin America: accomplishments and challenges on treatment and prevention. Curr Opin HIV AIDS.

[17] van Griensven F, Mock PA, Benjarattanaporn P, Premsri N, Thienkrua W, Sabin K (2018). Estimating recent HIV incidence among young men who have sex with men: reinvigorating, validating and implementing Osmond’s algorithm for behavioral imputation. PLoS ONE.

[18] Lane T, Osmand T, Marr A, Struthers H, McIntyre JA, Shade SB (2016). Brief report: high HIV incidence in a South African community of men who have sex with men: results from the mpumalanga Men’s study, 2012–2015. J Acquir Immune Defic Syndr.

[19] Hallett TB (2011). Estimating the HIV incidence rate: recent and future developments. Curr Opin HIV AIDS.

[20] Marcus U, Voss L, Kollan C, Hamouda O (2006). HIV incidence increasing in MSM in Germany: factors influencing infection dynamics. Euro Surveill.

[21] UNAID. 90–90–90—An ambitious treatment target to help end the AIDS epidemic. UNAIDS / JC2684 (English original, October 2014). https://www.unaids.org/en/resources/documents/2017/90-90-90.

[22] Hernandez I, Reina-Ortiz M, Johnson A, Rosas C, Sharma V, Teran S (2017). Risk factors associated with HIV among men who have sex with men (MSM) in Ecuador. Am J Mens Health.

[23] Brignol S, Kerr L, Amorim LD, Dourado I, Brignol S, Kerr L (2016). Factors associated with HIV infection among a respondent-driven sample of men who have sex with men in Salvador. Brazil Rev Bras Epidemiol.

[24] Chamberlain N, Mena LA, Geter A, Crosby RA (2017). Is sex with older male partners associated with higher sexual risk behavior among young black MSM?. AIDS Behav.

[25] Khosropour CM, Dombrowski JC, Swanson F, Kerani RP, Katz DA, Barbee LA (2016). Trends in serosorting and the association with HIV/STI risk over time among men who have sex with men. J Acquir Immune Defic Syndr.

[26] Redoschi BRL, Zucchi EM, dos Santos Barros CR, Paiva VSF, Redoschi BRL, Zucchi EM (2017). Routine HIV testing in men who have sex with men: from risk to prevention. Cad Saúde Pública.

[27] Hess KL, Crepaz N, Rose C, Purcell D, Paz-Bailey G (2017). Trends in sexual behavior among men who have sex with men (MSM) in high-income countries, 1990–2013: a systematic review. AIDS Behav.

[28] Del Pino HE, Harawa NT, Liao D, Moore AA, Karlamangla AS (2018). Age and age discordance associations with condomless sex among men who have sex with men. AIDS Behav.

[29] Banandur P, Rajaram SP, Mahagaonkar SB, Bradley J, Ramesh BM, Washington RG (2011). Heterogeneity of the HIV epidemic in the general population of Karnataka state, south India. BMC Public Health.

